# The Relationship Between Thyroid Function or Subclinical Hypothyroidism in Early Pregnancy and Risk of Low Birth Weight and Small for Gestational Age of the Offspring: A Multicentre Prospective Cohort Study

**DOI:** 10.7150/ijms.121837

**Published:** 2026-01-01

**Authors:** Juan Li, Minhui Hu, Rong Zhao, Shuanghua Xie, Shaofei Su, Enjie Zhang, Shuangying Li, Zhan Li, Jianhui Liu, Hao Xing, Ruixia Liu, Aris T. Papageorghiou, Chenghong Yin

**Affiliations:** 1 Department of Central Laboratory, Beijing Obstetrics and Gynecology Hospital, Capital Medical University, Beijing Maternal and Child Health Care Hospital, Beijing 100026, China.; 2 Department of Obstetrics, Beijing Obstetrics and Gynecology Hospital, Capital Medical University, Beijing Maternal and Child Health Care Hospital, Beijing 100026, China.; 3 Nuffield Department of Women's and Reproductive Health, University of Oxford, The Women's Centre, John Radcliffe Hospital, Oxford, UK.

**Keywords:** thyroid function, thyroid-stimulating hormone, free thyroxine, low birth weight, small for gestational age

## Abstract

**Objective:** To examine how maternal thyroid-stimulating hormone (TSH), free thyroxine (FT4) and thyroid peroxidase antibody (TPOAb) status in early pregnancy relate to low birth weight (LBW) or small for gestational age (SGA) outcomes.

**Methods:** This prospective cohort analysis utilized data from 125,365 singleton pregnancies in the China Birth Cohort Study (2018-2022), with participants enrolled at 6-13^+6^weeks gestation from 9 tertiary hospitals. The potential associations among maternal thyroid functional indices, spectrum of thyroid dysfunction, and adverse neonatal outcomes (LBW/SGA) were statistically evaluated employing generalized linear mixed modeling techniques. Besides, to verify the consistency of these findings, we conducted comprehensive subgroup analyses across multiple demographic and clinical strata.

**Results:** Among the final 86,015 eligible participants, LBW and SGA occurred in 3.18% (n=2,731) and 3.56% (n=3,060), respectively. After adjusting for maternal and neonatal characteristics, analyses revealed significant negative associations between circulating maternal thyroid hormone levels and offspring birth weight measurements (per 1 mIU/L increase in TSH: β = -5.62, 95% CI: -7.29 to -3.95, *P* < 0.001; per 1 pmol/L increase in FT4: β = -1.43, 95% CI: -2.21 to -0.65, *P* < 0.001). First-trimester subclinical hypothyroidism (SCH) was associated with increased risks of both LBW (aOR = 1.29, 95% CI: 1.04-1.59; *P* = 0.021) and SGA (aOR = 1.18, 95% CI:1.01-1.38; *P* = 0.037). Women in the highest TSH quintile had 20% higher LBW risk (aOR = 1.20, 95% CI: 1.02-1.41; *P* = 0.028) and 16% higher SGA risk compared to the lowest quintile (aOR = 1.16, 95% CI: 1.03-1.30;* P* = 0.012). The associations of TSH and FT4 with LBW and SGA were consistent across all subgroups.

**Conclusions:** Elevated maternal TSH, elevated FT4 (even within high-normal ranges), and SCH in early pregnancy serve as significant risk indicators for LBW and SGA.

## Introduction

In pregnancy, thyroid hormones are essential as they affect placental function, fetal development, and the expression of neuropeptides associated with parturition 1. Thyroid pathologies—are among the most prevalent endocrine conditions in pregnancy and include subclinical hypothyroidism (SCH) —the most common thyroid dysfunction affecting 3.5% of all women 2, hyperthyroidism, and hypothyroidism. Abnormal maternal thyroid status may contribute to several adverse perinatal outcomes, and has been associated with gestational diabetes, hypertensive disorders, miscarriage, preterm birth, neonatal intensive care unit (NICU) admission, low APGAR scores, low birth weight (LBW) and small for gestational age (SGA) 3-9. Both LBW and SGA— pose a significant global health burden as they collectively affect an estimated 20 million neonates annually 10 and are linked to elevated likelihood of late-onset noncommunicable conditions 11-13.

Nevertheless, the potential link between maternal thyroid abnormalities and compromised fetal growth outcomes (LBW/SGA) has yet to reach consensus in the research community. Some studies suggest that elevated maternal thyrotropin (TSH) and free thyroxine (FT4) levels are inversely correlated with birth weight, so that higher FT4 in early pregnancy increases the likelihood of LBW 11, 14. Conversely, other studies report no significant association 15, 16. Similarly, first-trimester TSH levels between 4-10mIU/L were associated with significantly increased risks of SGA 17 in some but not other studies 18. This existing evidence is limited by small sample sizes and methodological inconsistencies in defining thyroid reference intervals 19.

To address these gaps in knowledge which are in part due to small study sizes, methodological inconsistencies and varying definitions of thyroid reference intervals, this large population-based cohort investigation was embedded within the China Birth Cohort Study (CBCS) 20. The core objective of this study is to systematically examine the nature and magnitude of correlations between maternal thyroid biomarker profiles during the specific gestational window of 6 to 13^+6^ weeks—specifically, TSH concentrations, FT4 levels, and thyroid peroxidase antibody (TPOAb) positivity—and adverse birth outcomes in offspring, including LBW and SGA. This investigation seeks to provide a foundational evidence base for research exploring the relationship between thyroid function management during pregnancy and fetal health outcomes.

## Materials and Methods

### Subjects

This multicentre prospective cohort study utilized data from the CBCS 20, analyzing 125,365 pregnant women who received first-trimester prenatal care (starting at a gestational age 6-13^+6^ weeks) across 9 hospitals between February 2018 and December 2022. All participants underwent first-trimester thyroid function testing and subsequently delivered live-born singletons. We excluded: (1) ultrasound-confirmed non-singleton pregnancy in the first trimester; (2) Pregnancies conceived through assisted reproductive technology (ART), such as in vitro fertilization, intracytoplasmic sperm injection, or embryo transfer; (3) Prior diagnoses of thyroid conditions, encompassing pre-pregnancy thyroid dysfunction, surgical removal of the thyroid, thyroiditis, as well as cystic or neoplastic thyroid lesions; (4) Missing thyroid function values(FT4, TSH, TPOAb); (5)Preconception intake of thyroid-modulating drugs (i.e. L-T4, propylthiouracil, methimazole); (6) Missing data on neonatal birth weight; (7) Unknown fetal sex; (8) Gestational age at birth outside 28-41^+6^ weeks. After screening and eligibility assessment, 86,015 pregnant women were enrolled in this investigation. Based on prior evidence showing an odds ratio (OR) of 1.16-1.24^11^, ^21^ between thyroid function and SGA, and a background SGA prevalence of 3.56% in our population, a sample size of 9,448-19,847 was estimated to provide sufficient power (α=0.05, δ=0.10). The research protocol was approved by the institutional ethics committee (Beijing Obstetrics and Gynecology Hospital, Capital Medical University; Approval No. 2018-KY-003-02), and written informed consent was properly executed by each participant before any study procedures.

### Data collection

Through standardized questionnaire administration, demographic profiles were established 20. Baseline data included maternal age, pre-pregnancy BMI, ethnic group, smoking, alcohol consumption status and obstetric history. Educational attainment was classified into three levels: primary school or lower, high school or lower, and college or above. Annual household income was stratified as < 100,000 CNY, 100,000-400,000 CNY, or > 400,000 CNY. Binary variables (yes/no) were used to document pre-existing medical conditions (hypertension and diabetes) and health behaviours (smoking and alcohol consumption). Additionally, we collected data on pre-pregnancy thyroid disease history, thyroid-related medication status, gestational weeks at parturition and neonatal sex. Through digital means, all records are consistently gathered and kept up to date.

Blood collection occurred at the initial prenatal visit at 6-13^+6^ weeks of gestation, following fasting for 8-10h overnight. Serum levels of TSH, FT4, and TPOAb were measured using an electrochemiluminescence immunoassay system. The manufacturer's recommended threshold was applied, with TPOAb concentrations ≥ 60 IU/L considered positive. International consensus (e.g., ATA guidelines) recommends the adoption of trimester-specific reference ranges derived from healthy pregnant women who have sufficient iodine levels, no thyroid dysfunction, and negative TPOAb status 22. All participants were recruited from regions that have implemented universal salt iodization since 1995, where the general population maintains replete iodine nutrition without widespread deficiency or excess. Following the predefined inclusion and exclusion criteria (see Methods), we further excluded individuals who tested positive for TPOAb. Euthyroidism was defined as serum TSH and FT4 levels falling within the 5th-95th percentile ranges (TSH: 0.13-3.60 mU/L; FT4: 11.35-19.62 pmol/L). This approach accounts for potential sample storage effects and is consistent with reference ranges established in previous studies 19, 23. Thyroid dysfunction was classified as overt hyperthyroidism (TSH < 5.0th percentile with FT4 > 95.0th percentile), overt hypothyroidism (TSH > 95.0th percentile with FT4 < 5.0th percentile), subclinical hyperthyroidism (TSH < 5.0th percentile with normal FT4), subclinical hypothyroidism (TSH > 95.0th percentile with normal FT4), and isolated hypothyroxinemia (FT4 < 5.0th percentile with normal TSH). All classifications were based on the established reference ranges, with normal function defined as both TSH and FT4 falling within the 5.0th-95.0th percentiles.

LBW was defined as birth weight less than 2,500g 24, SGA was classified as a birth weight below the 10th percentile for sex and gestational age based on INTERGROWTH-21st standards 25.

### Statistical analysis

Data were presented as mean ± SD (normally distributed), median (IQR) (non-normal), or frequencies (%) (categorical). Group comparisons (LBW vs. non-LBW; SGA vs. non-SGA) used t-tests, Mann-Whitney U tests, or χ²/Fisher's exact tests as appropriate. We used linear regression to assess the relationship between maternal thyroid function and offspring birth weight, while employing multivariate logistic regression to evaluate the associations of maternal thyroid hormone levels and diseases with the risks of LBW and SGA. A restricted cubic spline model was further applied to characterize the exposure-response relationships more flexibly. For these analyses, TSH and FT4 were treated as continuous variables and also categorized into quintiles to assess potential linear trends.

To examine the consistency of the links between maternal thyroid hormone levels (TSH/FT4) and poor neonatal outcomes (LBW and SGA), we conducted stratified analyses across clinically relevant subgroups. Maternal age (< 35 vs. ≥ 35 years), pre-pregnancy BMI (< 25 vs. ≥ 25 kg/m²), parity (nulliparous vs. multiparous), neonatal sex (male vs. female), and TPOAb positive (yes vs.no) were considered as potential effect modifiers. Interaction P-values were computed to assess whether these variables significantly influenced the observed relationships.

Analyses were performed using SPSS 25.0 and RStudio4.0.3.

## Results

### Study population enrollment and exclusion

**Figure 1:**
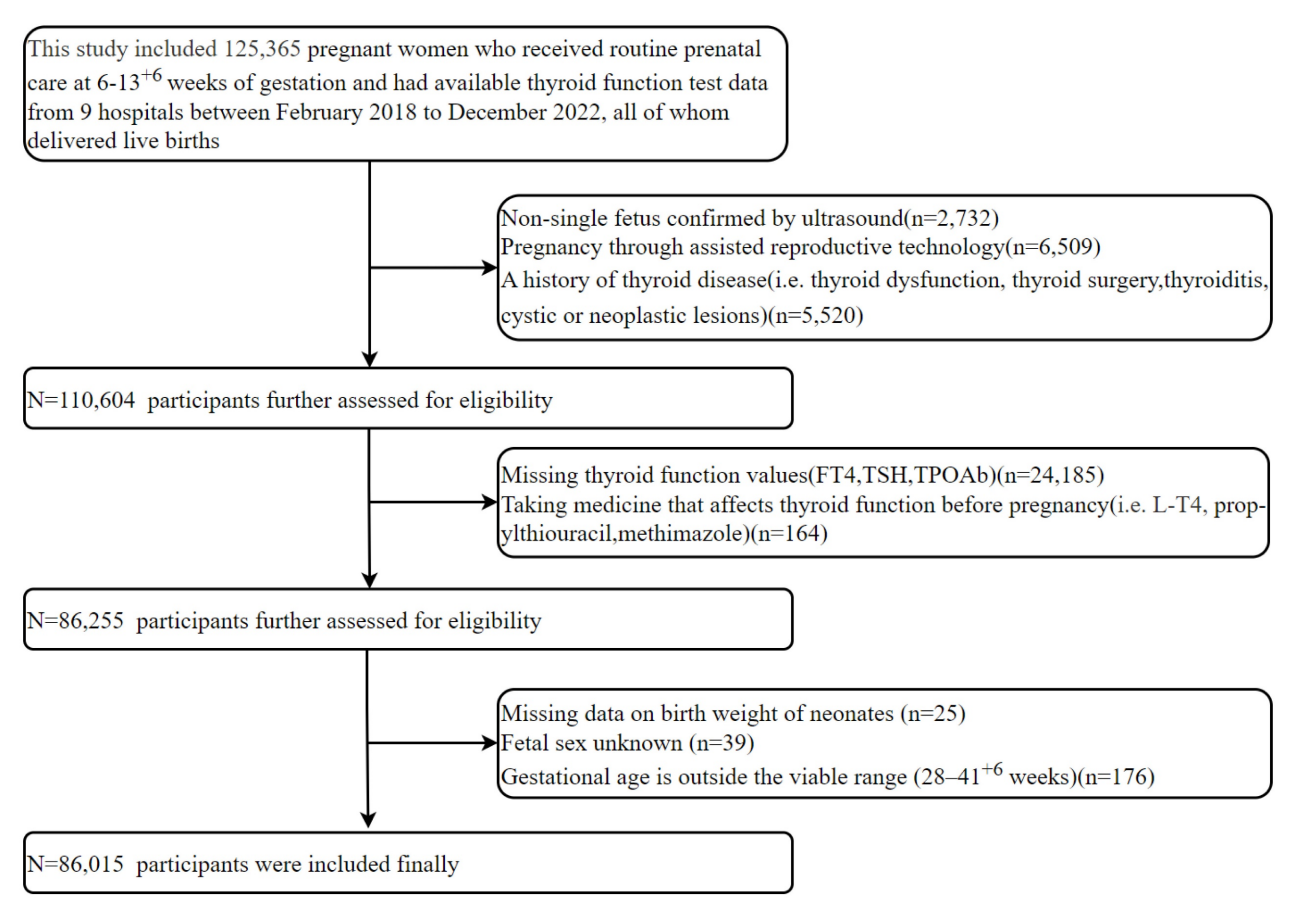
Flow chart of study participants selection.

After applying exclusion criteria to the initial 125,365 women, 86,015 were included in the final analysis (Figure 1), with exclusions as follows: non-singleton pregnancy (n = 2,732); conception by ART (n = 6,509); preexisting thyroid conditions (n = 5,520); missing thyroid function values (n = 24,185); use of thyroid medications (n=164); missing neonatal birth weight data (n = 25); unknown fetal sex (n = 39); and non-viable gestational age (n = 176). This rigorous process aimed to minimize bias and enhance internal validity.

### Baseline characteristics of the study population

The study cohort included 86,015 eligible participants, comprising 2,731 LBW cases (3.18%) and 3,060 SGA cases (3.56%), (Table 1). Table 1 demonstrates statistically significant disparities in maternal age, pre-conception BMI, annual family income, gestational age at delivery, and TSH levels between LBW and non-LBW groups, as well as between SGA and non-SGA groups. Mothers of LBW infants had higher rates of pre-existing hypertension (1.6% vs. 0.3%, *P* < 0.001), lower educational attainment (high school or below: 12.9% vs. 10.3%, *P* < 0.001). Furthermore, a higher proportion of LBW infants were female (52.5% vs. 48.3%, *P* < 0.001). For SGA, significant associations were found with maternal alcohol consumption (3.3% vs. 4.0%, *P* = 0.031), multiparity (65.9% vs. 54.4%, *P* < 0.001), and TPOAb positivity (7.2% vs. 8.2%, *P* = 0.039). However, no meaningful linkages were observed between LBW/SGA and maternal race, maternal smoking, previous diabetes or FT4 levels.

### Association between first-trimester thyroid hormone levels and neonatal birth weight

As shown in Table 2, crude analyses demonstrated that each 1 mIU/L increase in maternal TSH was inversely associated with birth weight (β = -5.00 g, 95% CI: -7.01 to -2.99,* P* < 0.001). A similar inverse association was observed for each 1 pmol/L increase in maternal FT4 (β = -3.20 g, 95% CI: -4.15 to -2.26, *P* < 0.001). These inverse relationships remained significant after adjusting for maternal age, pre-conception BMI, maternal smoking/drinking behaviors, educational attainment, annual family income, parity, pre-existing hypertension or diabetes, gestational age at delivery, neonatal sex, TPOAb positive and regional clustering effects. Importantly, the adjusted effect sizes remained consistent (per 1 mIU/L increase in TSH: β = -5.62, 95% CI: -7.29 to -3.95, *P* < 0.001; per 1 pmol/L increase in FT4: β = -1.43, 95% CI: -2.21 to -0.65, *P* < 0.001). In contrast, regardless of adjustment for covariates, maternal TPOAb status demonstrated no statistically significant effect on infant birth weight.

### Correlation between early-pregnancy maternal thyroid hormone levels, thyroid disorders and the risk of LBW or SGA

Adjusted models revealed a significant link between maternal thyroid status and neonatal complications. As shown in Table 3**,** each 1-unit increase in FT4 was associated with a 2% higher risk of LBW (aOR 1.02, 95% CI 1.00-1.03, *P* = 0.033), while each 1-unit TSH increase was associated with a 2% greater SGA risk (aOR 1.02, 95% CI 1.01-1.04, *P* = 0.012), after adjustment for confounders. Subclinical hypothyroidism significantly increased the odds of both LBW (aOR 1.29, 95% CI 1.04-1.59, *P* = 0.021) and SGA (aOR 1.18, 95% CI 1.01-1.38, *P* = 0.037) compared to euthyroidism. TPOAb positivity showed no significant associations in any model.

### Stratified analysis of LBW/SGA risk by maternal TSH and FT4 Levels

As can be seen in Table 4, in the quintile analysis of maternal FT4 levels, no individual quintile was associated with a significantly increased risk of LBW compared with the lowest quintile after full adjustment for confounders. However, a statistically significant linear trend was observed across the FT4 quintiles (*P* for trend = 0.033). For TSH levels, women in the highest quintile had a modestly increased risk of both SGA and LBW compared with those in the lowest quintile. The adjusted ORs were 1.16 (95% CI: 1.03-1.30; *P* = 0.012) for SGA and 1.20 (95% CI: 1.02-1.41; *P* = 0.028) for LBW, representing 16% and 20% increased risks, respectively. Additionally, a significant dose-response relationship was evident for SGA, as indicated by a significant trend across the quintiles (*P* for trend = 0.012).

**Figure 2:**
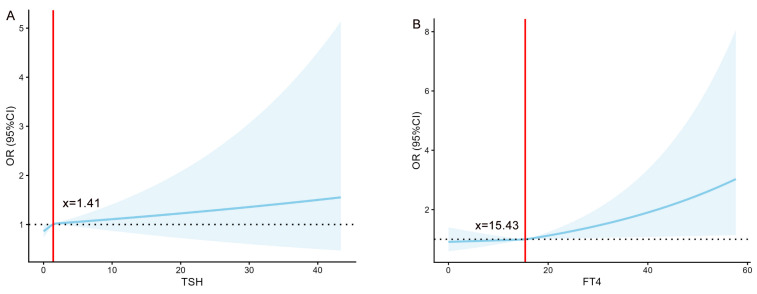

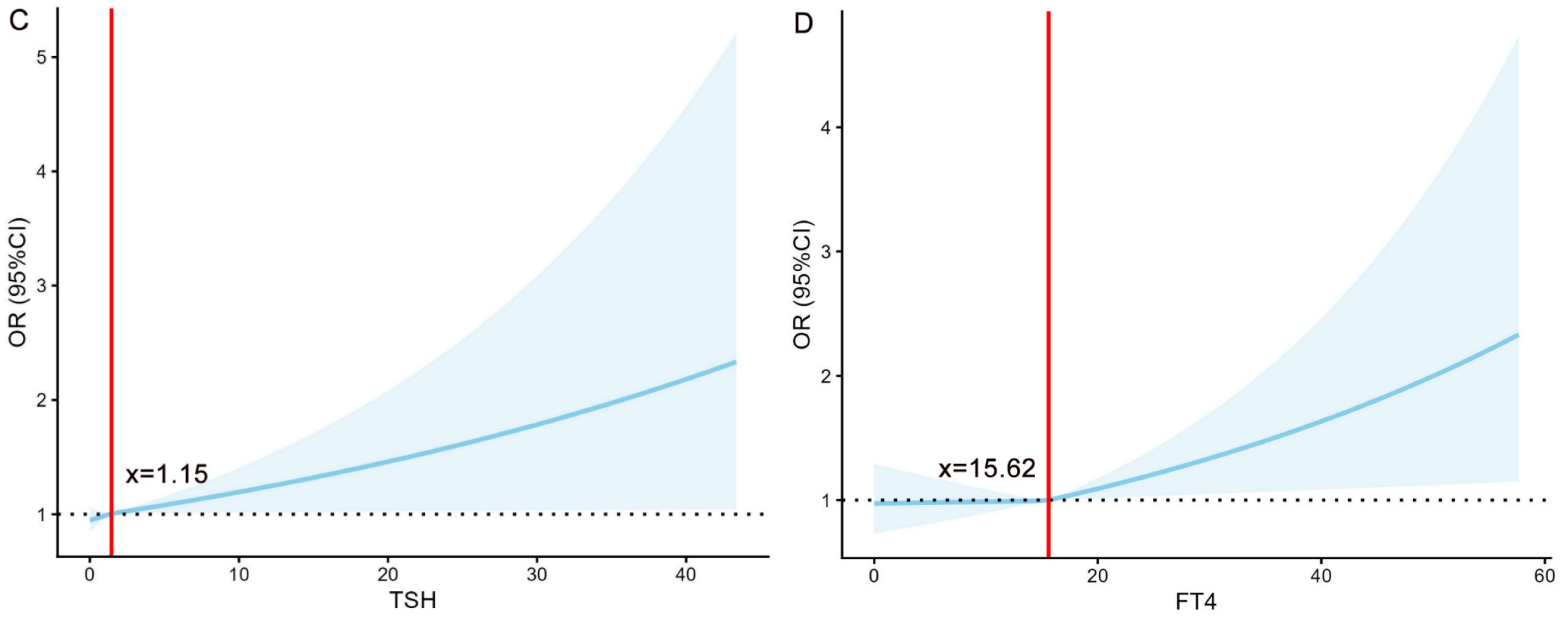
The association of TSH (mIU/L) /FT4 (pmol/L) with the risk of LBW/SGA. (A) The relationship between TSH and the risk of LBW. (B) The relationship between FT4 and the risk of LBW. (C) The relationship between TSH and the risk of SGA. (D) The relationship between FT4 and the risk of SGA. FT4, free thyroxine; TSH, thyroid-stimulating Hormone; LBW, low birth weight; SGA, small for gestational age; OR, odds ratio.

To quantify the associations of TSH and FT4 with the risks of LBW and SGA, this study employed restricted cubic spline models for analysis (Figure 2). The analysis revealed significant nonlinear associations between TSH and SGA risk, as well as between FT4 and LBW risk (all *P* < 0.001). Specifically, the risks remained stable below specific thresholds but increased markedly once these thresholds were exceeded, indicating that elevated levels of both FT4 and TSH serve as important warning signals for an increased risk of both LBW and SGA.

### Subgroup analysis

Subgroup analyses (stratified by maternal age, pre-pregnancy BMI, parity, fetal sex, and TPOAb positivity) demonstrated that the positive association between elevated TSH (≥ 2.33 mIU/L) and the risks of both LBW and SGA was consistent across all subgroups, with no significant interaction effects observed (Figure S1 and S3). While the association between FT4 and LBW initially appeared stronger in TPOAb-positive women, this difference was no longer significant after false discovery rate (FDR) correction, suggesting a potential false-positive finding (Figure S2). Similarly, the association between FT4 and SGA remained consistent across all subgroups (Figure S4).

## Discussion

Our study identified independent dose-response relationships between elevated first-trimester maternal TSH and FT4 levels with reduced neonatal birth weight. Subclinical hypothyroidism was associated with significantly increased risks of both LBW and SGA compared to euthyroidism. Furthermore, the associations of TSH and FT4 with LBW/SGA demonstrated nonlinear threshold effects. Notably, the positive association between elevated TSH (≥ 2.33 mIU/L) and the risks of LBW/SGA remained consistent across all subgroups, underscoring the robustness of this relationship.

This study demonstrates methodological rigor through several key strengths. First, the large multicentre cohort comprising 86,015 pregnant women from 9 hospitals provides robust statistical power while minimizing selection bias. Second, standardized thyroid function assessments during the first trimester (6-13^+6^ weeks) were undertaken to ensure measurement precision. Third, our analytical approach incorporates comprehensive covariate adjustment and clinically stratified analyses, strengthening causal inference while maintaining clinical relevance. We believe that these methodological features ensure a rigorous foundation for evaluating the effects of thyroid function on newborn size. However, as any study, ours had some limitations. Thyroid function was assessed only at a single early-pregnancy timepoint (6-13⁺⁶ weeks); while this precludes evaluation of dynamic changes throughout gestation, longitiduonal assessment was not feasible in such a large cohort. Furthermore, urinary iodine concentration (UIC) was not systematically measured. To address the latter, we restricted the population to regions with universal salt iodization to minimize confounding. Nevertheless, the multicenter design and stratified analyses support the generalizability of our findings. Future studies incorporating repeated thyroid measurements and individual UIC data would better delineate temporal relationships and independent effects on perinatal outcomes.

Our study revealed a 3.18% incidence of LBW, aligning with previous Chinese reports (2.77-6.10%) 26, 27. Current evidence indicates that thyroid dysfunction affects fetal growth. Studies demonstrate that elevated FT4 correlates with reduced birth weight 28, 29, consistent with findings in thyrotoxicosis showing a higher LBW incidence (23.7% vs. 17.7%) 30. Recent first-trimester data also reveal a similar inverse relationship between FT3 and birth weight 31. Although findings regarding TSH remain inconsistent, the persistent association between thyroid hormones and birth weight across trimesters 32 collectively suggests a continuous inhibitory effect on fetal growth throughout pregnancy.

In addition, the 21% elevated SGA risk in the highest TSH quintile supports previously reported links between SCH and fetal growth restriction 33, 34. The 2017 ATA guidelines recommend TPOAb testing to assess thyroid autoimmunity due to its high prevalence 22. While TPOAb positivity is a recognized predictor of gestational thyroid dysfunction 35-37, our study found no independent association between TPOAb status and reduced birth weight.

Restricted cubic spline analyses revealed nonlinear relationships between TSH/FT4 levels and LBW/SGA risk (*P* < 0.05). Although quintile analyses suggested monotonic trends, spline models demonstrated accelerated risk elevation at upper-normal hormone levels, indicating threshold effects that categorical analyses may overlook. While the continuous effects of thyroid hormones on birth weight are clinically insignificant (1.43 g per FT4 unit; 5.62 g per TSH unit), our key finding relates to categorical outcomes. When TSH exceeds 2.33 mIU/L, risks of both LBW (aOR = 1.16) and SGA (aOR = 1.20) increase significantly. This threshold-based association supports the 2.5 mIU/L upper limit recommended by major guidelines 38, 39 and justifies targeted monitoring for at-risk individuals. Indeed, early thyroid screening remains clinically meaningful despite minimal per-unit weight effects.

It should be noted that some studies have reported no significant link between thyroid dysfunction and LBW 5, 16, 40, while others identified TPOAb positive in the first trimester as an independent predictor (OR = 7.76, 95% CI: 1.23-48.86,* P* = 0.029) 8, 36, 37 —a conclusion contrasting with our results, which showed no such association. Moreover, a newly published prospective cohort analysis demonstrated the absence of any significant connection between SCH (whether presenting in the first or third gestational trimester) and the likelihood of delivering SGA neonates., regardless of TPOAb status 41. We believe these inconsistencies may reflect methodological differences in gestational timing, statistical power, and confounding adjustment across studies.

The efficacy of levothyroxine (L-T4) therapy for SCH in pregnancy remains controversial. While meta-analyses demonstrate significant benefits—one showing a 65% reduction in LBW risk (RR = 0.35, 95% CI: 0.18-0.69) 42 and another reporting a 92% risk reduction (OR = 0.08, 95% CI: 0.01-0.51) 43—contradictory evidence exists. Retrospective studies by Luo et al. 44and Han et al. 45 found either no improvement in neonatal outcomes or persistent LBW risk despite treatment (aOR = 2.11, 95% CI: 1.42-3.13). These discrepancies underscore the critical need for better patient stratification. Consequently, future randomized trials should validate whether personalized L-T4 therapy based on screening can effectively reduce LBW and SGA incidence.

Several mechanisms may explain our findings. First, elevated maternal FT4 levels in early pregnancy could increase placental vascular resistance, altering hemodynamics and impairing placental functions such as nutrient supply and waste elimination, ultimately contributing to LBW 1, 28, 46. This effect may be mediated via placental TSH receptors or modulated by placental deiodinase activity 1, 47. Second, high FT4 levels may accelerate maternal catabolism, leading to chronic energy deficiency and reduced birth weight 48. Third, maternal thyroid dysfunction (including SCH) might impair fetal vascular development, while deficiencies in essential metals could disrupt thyroid hormone homeostasis, both potentially increasing susceptibility to LBW and SGA 49, 50. Coming research efforts should pursue two key objectives: clarifying the causal pathways and testing targeted prevention approaches.

## Conclusion

Our findings reinforce that maternal thyroid function, even within subclinical ranges, are significantly associated with fetal growth outcomes. Collectively, our results highlight the potential clinical utility of thyroid monitoring during gestation, especially among women exhibiting FT4 concentrations in the high-normal range or elevated TSH levels.

## Supplementary Material

Supplementary figures.

## Figures and Tables

**Table 1:** General characteristics among the study population

Demographics	Total (n=86,015)	LBW (n=2731)	Non-LBW (n=83,284)	SGA (n=3060)	Non-SGA (n=82,955)
Age(years), n (%) **^a, b^**	86,015				
<35	74,397 (86.5)	2250(82.4)	72,147 (86.6)	2756(90.1)	71,641 (86.4)
≥35	11,618(13.5)	481(17.6)	11,137(13.4)	304(9.9)	11,314 (13.6)
Pre-pregnancy BMI, n (%)**^ a, b^**					
<25	75,637 (87.9)	2318 (84.9)	73,319(88.0)	2782 (90.9)	72,855(87.8)
≥25	10,378 (12.1)	413(15.1)	9965(12.0)	278(9.1)	10,100(12.2)
Ethnic, n (%)					
Han	81,729(95.0)	2589(94.8)	79,140(95.0)	2908 (95.0)	78,821(95.0)
Others	4286 (5.0)	142(5.2)	4144 (5.0)	152(5.0)	4134(5.0)
Smoking, n (%)					
Yes	170(0.2)	8(0.3)	162(0.2)	10(0.3)	160(0.2)
No	85,845 (99.8)	2723 (99.7)	83,122(99.8)	3050 (99.7)	82,795(99.8)
Drinking, n (%)**^ b^**					
Yes	3456(4.0)	102(3.7)	3354(4.0)	100(3.3)	3356(4.0)
No	82,559(96.0)	2629(96.3)	79,930 (96.0)	2960(96.7)	79,599 (96.0)
Parity, n (%) **^b^**					
Nullipara	38,896(45.2)	1218 (44.6)	37,678(45.2)	1043 (34.1)	37,853 (45.6)
Multipara	47,119 (54.8)	1513(55.4)	45,606 (54.8)	2017(65.9)	45,102 (54.4)
Maternal education, n (%) **^a^**					
Primary school or lower	105(0.1)	5 (0.2)	100 (0.1)	5 (0.2)	100 (0.1)
High school or lower	8791(10.2)	348 (12.7)	8443(10.2)	305 (10.0)	8486 (10.2)
College or above	77,119(89.7)	2378 (87.1)	74,741(89.7)	2750 (89.8)	74,369(89.7)
Household annual income, n (%)**^ a, b^**					
<100,000,CNY	18,218(21.2)	681(25.0)	17,537 (21.1)	770 (25.2)	17,488 (21.0)
100,000, CNY-400,000, CNY	54,560(63.4)	1670 (61.1)	52,890(63.5)	1881(61.5)	52,679(63.5)
>400,000, CNY	13,237 (15.4)	380 (13.9)	12,857(15.4)	409 (13.3)	12,828(15.5)
Pre-hypertension disease, n (%) **^a^**					
Yes	259 (0.3)	45(1.6)	214 (0.3)	12(0.4)	247 (0.3)
No	85,756 (99.7)	2686(98.4)	83,070(99.7)	3048 (99.6)	82,708 (99.7)
Previous diabetes, n (%)					
Yes	271(0.3)	14 (0.5)	257 (0.3)	6 (0.2)	265 (0.3)
No	85,744(99.7)	2717 (99.5)	83,027 (99.7)	3054(99.8)	82,690 (99.7)
Gestational age at delivery, mean ± SD**^ a, b^**	38.86±1.44	34.97±2.69	38.98±1.18	38.74±1.62	38.86±1.43
Neonatal gender, n (%)^**a**^					
Male	44,354(51.6)	1298 (47.5)	43,056(51.7)	1583(51.7)	42,771(51.6)
Female	41,661 (48.4)	1433(52.5)	40,228(48.3)	1477(48.3)	40,184(48.4)
TSH, mU/L, mean ± SD**^ a, b^**	1.58±1.50	1.68±1.50	1.58±1.50	1.67±1.70	1.58±1.49
FT4, pmol/L, mean ± SD	15.36±3.18	15.43±3.33	15.36±3.18	15.41±3.54	15.36±3.17
TPOAb positive, n (%)**^ b^**					
Yes (≥60U/L)	7050(8.2)	227 (8.3)	6823 (8.2)	220 (7.2)	6830 (8.2)
No (<60U/L)	78,965 (91.8)	2504(91.7)	76,461(91.8)	2840(92.8)	76,125(91.8)

^a^ Significant differences were observed between the LBW and non-LBW groups (*P* < 0.05) ^b^ Significant differences were observed between the SGA and non-SGA groups (*P* < 0.05) Abbreviation: LBW, low birth weight; SGA, small for gestational age; BMI, body mass index; SD, standard deviation; TSH, thyroid-stimulating hormone; FT4, free thyroxine; TPOAb, thyroid peroxidase antibody.

**Table 2:** Association of thyroid function in early pregnancy with neonatal birth weight

	Crude Modelβ (95% CI)	Adjusted Modelβ (95% CI)
neonatal birth weight		
TSH	**-5.00 (-7.01, -2.99)**	**-5.62(-7.29, -3.95)**
FT4	**-3.20(-4.15, -2.26)**	**-1.43(-2.21, -0.65)**
TPOAb positive	-2.70(-13.67, 8.26)	4.16(-13.20, 4.88)

*P* < 0.05 was considered to be statistically significant. Crude Modelβ: Unadjusted modelβ. Adjusted Modelβ: adjusted for maternal age, pre-pregnancy BMI, Smoking, Drinking, maternal education, household annual income, previous hypertension disease, previous diabetes, gestational age at delivery, neonatal sex, TPOAb positive and regional clustering effects. Abbreviation: TSH, thyroid-stimulating hormone; FT4, free thyroxine; TPOAb, thyroid peroxidase antibody.

**Table 3:** The association between maternal thyroid hormone levels and various thyroid disorders during early pregnancy and the risk of LBW or SGA.

	LBW			SGA	
	Adjusted Model ^a^ OR (95%CI)	P-value		Adjusted Model ^b^ OR (95%CI)	P-value
TSH	1.02(1.00- 1.05)	0.121		**1.02(1.01- 1.04)**	**0.012**
FT4	**1.02(1.00- 1.03)**	**0.033**		**1.01(1.00- 1.02)**	**0.046**
TPOAb positive	0.99(0.83- 1.19)	0.941		0.89(0.77-1.02)	0.100
Euthyroidism ^c^	Ref.			Ref.	
Overt hyperthyroidism	1.06(0.76- 1.48)	0.732		1.21(0.96- 1.54)	0.108
Overt hypothyroidism	1.03(0.50- 2.12)	0.928		0.91(0.54- 1.52)	0.706
Subclinical hyperthyroidism	0.77(0.55- 1.09)	0.144		0.84(0.65- 1.07)	0.155
Subclinical hypothyroidism	**1.29(1.04- 1.59)**	**0.021**		**1.18(1.01- 1.38)**	**0.037**
Isolated hypothyroxinemia	1.00(0.77- 1.30)	0.978		1.00(0.83- 1.19)	0.961

*P* < 0.05 was considered to be statistically significant. Adjusted Model ^a^: adjusted for maternal age, pre-pregnancy BMI, maternal education, household annual income, previous hypertension disease, gestational age at delivery, neonatal sex, parity × BMI interactions and regional clustering effects. Adjusted Model ^b^: adjusted for maternal age, pre-pregnancy BMI, Drinking, parity, household annual income, gestational age at delivery, TPOAb positive, parity × BMI interactions and regional clustering effects. ^c^ Defined as mothers with normal range (5^th^ to 95^th^ percentiles) for TSH and FT4 levels. Abbreviation: TSH, thyroid-stimulating hormone; FT4, free thyroxine; TPOAb, thyroid peroxidase antibody.

**Table 4:** Risk of LBW or SGA at different levels of TSH and FT4 in all enrolled women

	LBW		SGA	
	aOR (95%CI) ^a^	*P-*value	aOR (95%CI) ^b^	*P-*value
TSH (mIU/L)				
Q1(≤ 0.62)	Ref		Ref	
Q2(0.63-1.10)	0.98(0.83- 1.16)	0.826	1.06(0.94- 1.19)	0.346
Q3(1.11-1.59)	1.06(0.90- 1.25)	0.467	1.08(0.96- 1.21)	0.211
Q4(1.60-2.32)	1.10(0.93-1.29)	0.255	1.03(0.92-1.16)	0.586
Q5(≥2.33)	**1.20(1.02-1.41)**	**0.028**	**1.16(1.03-1.30)**	**0.012**
*P* for trend		0.121		**0.012**
FT4 (pmol/L)				
Q1(≤ 13.39)	Ref		Ref	
Q2(13.40-14.80)	1.09(0.92-1.29)	0.303	1.01(0.90-1.14)	0.822
Q3(14.81-15.96)	1.07(0.91-1.27)	0.411	0.99(0.88-1.12)	0.917
Q4(15.97-17.35)	1.16(0.98-1.37)	0.076	1.03(0.91-1.16)	0.642
Q5(≥17.36)	1.18(1.00-1.40)	0.053	1.06(0.94-1.20)	0.332
*P* for trend		**0.033**		**0.046**

*P* < 0.05 was considered to be statistically significant. aOR ^a^, Adjusted Model ^a^: adjusted for maternal age, pre-pregnancy BMI, maternal education, household annual income, previous hypertension disease, gestational age at delivery, neonatal sex, parity × BMI interactions and regional clustering effects. aOR ^b^, Adjusted Model ^b^: adjusted for maternal age, pre-pregnancy BMI, Drinking, parity, household annual income, gestational age at delivery, TPOAb positive, parity × BMI interactions and regional clustering effects. Abbreviation: LBW, low birth weight; SGA, small for gestational age; TSH, thyroid-stimulating hormone; FT4, free thyroxine.

## Abbreviations

LBW: low birth weight
SGA: small for gestational age
BMI: body mass index
CBCS: China Birth Cohort Study
FT4: free thyroxine
TPOAb: thyroid peroxidase antibody
TSH: thyroid-stimulating hormone
ART: Assisted Reproductive Technology
