# Supplementary material for: The Relationship Between Thyroid Function or Subclinical Hypothyroidism in Early Pregnancy and Risk of Low Birth Weight and Small for Gestational Age of the Offspring: A Multicentre Prospective Cohort Study
Source: Int J Med Sci. 2026 Jan 1;23(2):566–75. doi: 10.7150/ijms.121837 (PMC12825143; doi:10.7150/ijms.121837)
Supplement: Supplementary file 1 — Supplementary figures. [file ijmsv23p0566s1.pdf]

The Relationship Between Thyroid Function or Subclinical Hypothyroidism in Early Pregnancy and Risk of Low Birth Weight and Small for Gestational Age of The Offspring: A Multicentre Prospective Cohort Study

Juan Li,[1,] Minhui Hu,[1] Rong Zhao,[2] Shuanghua Xie,[1] Shaofei Su,[1] Enjie Zhang,[1] Shuangying Li,[1] Zhan Li,[1] Jianhui Liu,[1] Hao Xing,[1] Ruixia Liu,[1*] Aris T. Papageorghiou,[3*] Chenghong Yin.[1*]

[1] Department of Central Laboratory, Beijing Obstetrics and Gynecology Hospital, Capital Medical University, Beijing Maternal and Child Health Care Hospital, Beijing 100026, China

[2] Department of Obstetrics, Beijing Obstetrics and Gynecology Hospital, Capital Medical University, Beijing Maternal and Child Health Care Hospital, Beijing 100026, China

[3] Nuffield Department of Women′ s and Reproductive Health, University of Oxford, The Women's Centre, John Radcliffe Hospital, Oxford, UK.

* Corresponding author

Ruixia Liu

Email: liuruixia@ccmu.edu.cn

ORCID: 0000-0001-5835-4424

Tel.: +86-10-52277607. Address: No. 251 Yaojiayuan Road, Chaoyang District, Beijing 100026, China

Aris T. Papageorghiou

Email: aris.papageorghiou@wrh.ox.ac.uk

Tel.: +44(0)20 8725 0071. Address: John Radcliffe Hospital, Oxford, OX3 9DU, UK.

Chenghong Yin

Email: yinchh@ccmu.edu.cn

ORCID: 0000-0002-2503-3285

Tel.: +86-10-85968401. Address: No. 251 Yaojiayuan Road, Chaoyang District, Beijing 100026, China

Subgroup	OR (95% CI)	P-value	P for interaction	P for interaction [a]
**Age (years)**			**0.544**	**0.680**
<35	1.02(0.99-1.04)	0.113		
≥35	1.04(0.98-1.10)	0.067		
**Pre-pregnancy BMI (Kg/m2)**			**0.235**	**1.000**
< 25	1.03(1.01-1.06)	0.021		
≥25	0.99(0.93-1.05)	0.952		
**Parity**			**0.310**	**0.517**
Nullipara	1.04(0.99-1.09)	0.015		
Multipara	1.01(0.98-1.04)	0.387		
**Neonatal gender**			**1.000**	**1.000**
Male	1.02(0.97-1.06)	0.119		
Female	1.02(0.99-1.05)	0.103		
**TPOAb positive**			**0.259**	**0.648**
Yes(≥60U/L)	1.00(0.95-1.04)	0.001		
No(< 60U/L)	1.03(1.01-1.06)	0.204		

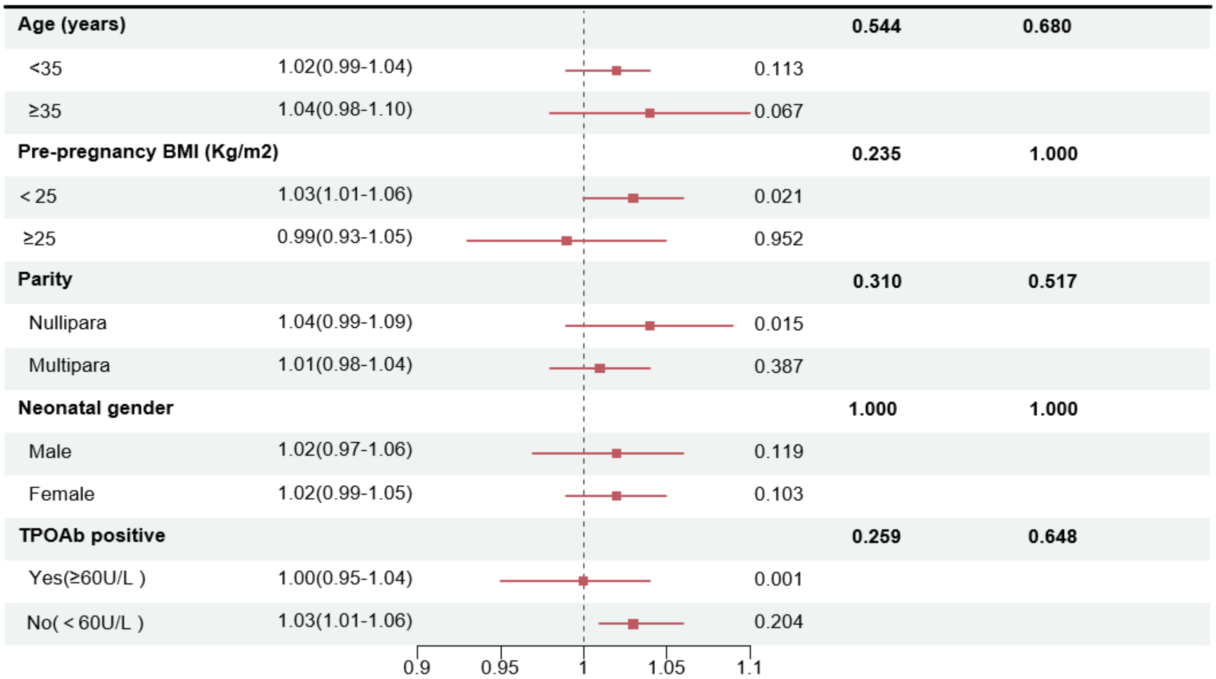

Figure S1 Association between TSH levels and LBW in different subgroup

Subgroup	HR (95% CI)	P−value	P for interaction	P for interaction [a]
**Age (years)**			**0.208**	**0.347**
<35	1.01(1.00−1.03)	0.223		
⩾35	1.04(1.00−1.09)	0.204		
**Pre−pregnancy BMI (Kg/m2)**			**0.470**	**0.588**
<25	1.02(1.00−1.04)	0.011		
⩾25	1.00(0.95−1.05)	0.726		
**Parity**			**0.166**	**0.415**
Nullipara	1.03(1.01−1.05)	0.079		
Multipara	1.01(0.99−1.03)	0.663		
**Neonatal gender**			**1.000**	**1.000**
Male	1.02(1.00−1.04)	0.409		
Female	1.02(1.00−1.04)	0.170		
**TPOAb positive**			**0.029**	**0.145**
Yes(⩾60U/L)	1.05(1.02−1.08)	0.887		
No(<60U/L)	1.01(0.99−1.03)	0.017		

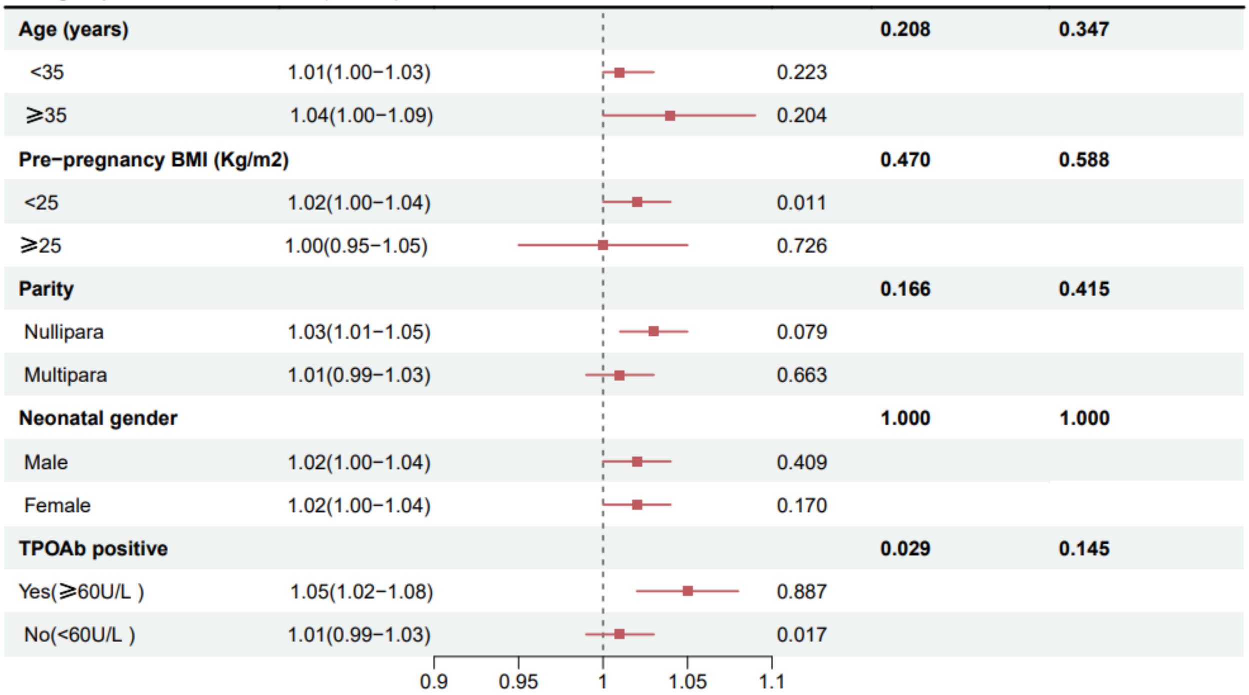

Figure S2 Association between FT4 levels and LBW in different subgroup

Subgroup	HR (95% CI)	P−value	P for interaction	P for interaction [a]
**Age (years)**			**0.332**	**0.415**
<35	1.02(1.01−1.04)	0.008		
⩾35	1.05(1.00−1.12)	0.059		
**Pre−pregnancy BMI (Kg/m2)**			**0.271**	**0.452**
<25	1.03(1.01−1.05)	0.001		
⩾25	0.98(0.90−1.07)	0.732		
**Parity**			**0.106**	**0.265**
Nullipara	1.06(1.02−1.11)	0.002		
Multipara	1.02(1.00−1.04)	0.059		
**Neonatal gender**			**0.066**	**0.33**
Male	1.04(1.02−1.06)	0.001		
Female	1.01(0.99−1.04)	0.273		
**TPOAb positive**			**0.588**	**0.588**
Yes(⩾60U/L)	1.02(0.99−1.05)	0.251		
No(<60U/L)	1.03(1.01−1.05)	0.005		

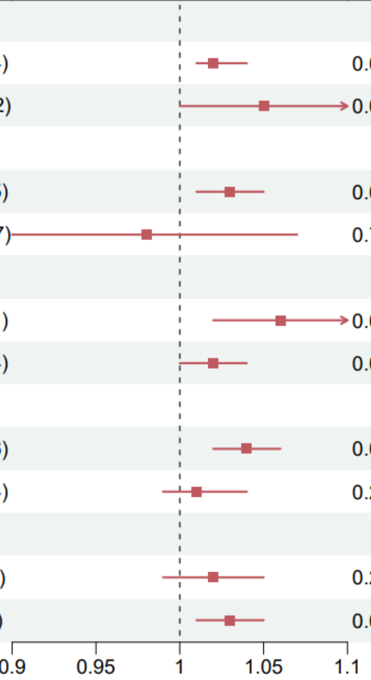

Figure S3 Association between TSH levels and SGA in different subgroup

Subgroup	HR (95% CI)	P−value	P for interaction	P for interaction [a]
**Age (years)**			**0.636**	**0.795**
<35	1.01(1.00−1.02)	0.248		
⩾35	1.00(0.96−1.04)	0.920		
**Pre−pregnancy BMI (Kg/m2)**			**0.160**	**0.800**
<25	1.01(1.00−1.02)	0.196		
⩾25	0.98(0.94−1.02)	0.387		
**Parity**			**0.435**	**0.725**
Nullipara	1.00(0.98−1.02)	0.873		
Multipara	1.01(0.99−1.02)	0.237		
**Neonatal gender**			**0.432**	**1.000**
Male	1.00(0.99−1.02)	0.639		
Female	1.01(0.99−1.03)	0.276		
**TPOAb positive**			**1.000**	**1.000**
Yes(⩾60U/L)	1.01(0.97−1.05)	0.647		
No(<60U/L)	1.01(0.99−1.02)	0.351		

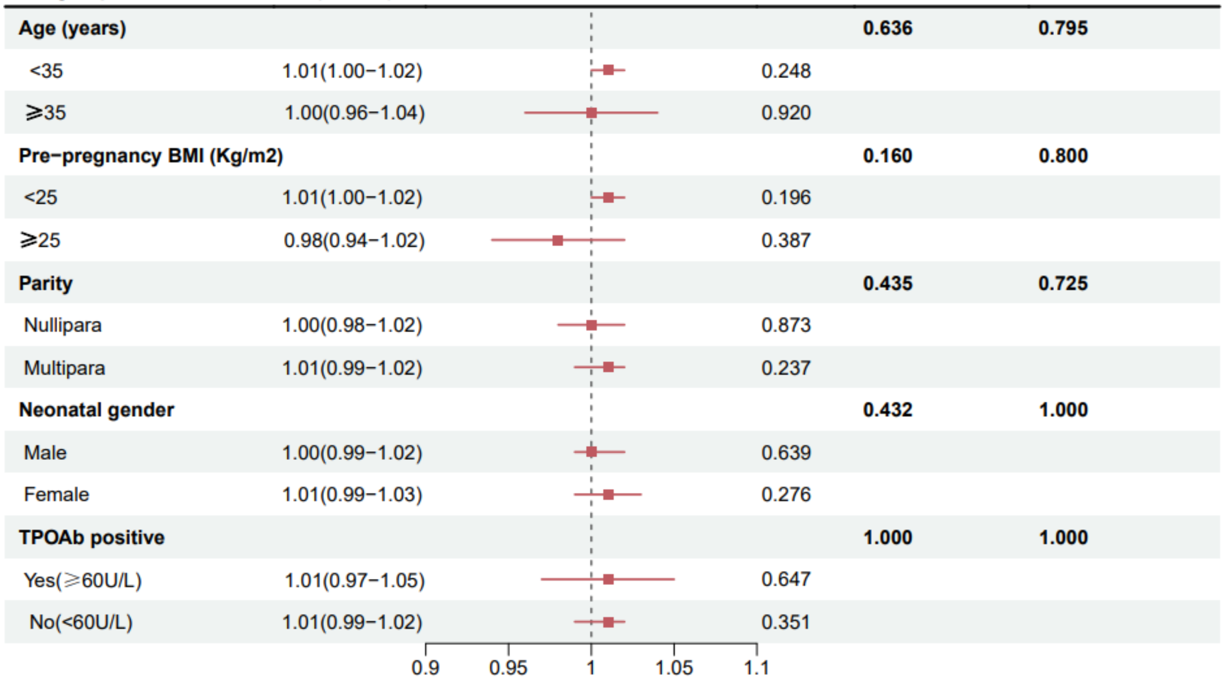

Figure S4 Association between FT4 levels and SGA in different subgroup
